# The Role of the Fiber/Bead Hierarchical Microstructure on the Properties of PVDF Coatings Deposited by Electrospinning

**DOI:** 10.3390/polym13030464

**Published:** 2021-02-01

**Authors:** Adrián Vicente, Pedro J. Rivero, José F. Palacio, Rafael Rodríguez

**Affiliations:** 1 Engineering Department, Campus de Arrosadía S/N, Public University of Navarre, 31006 Pamplona, Spain; 2 Campus de Arrosadía S/N, Institute for Advanced Materials and Mathematics (INAMAT2), Public University of Navarre, 31006 Pamplona, Spain; 3 Centre of Advanced Surface Engineering, AIN, 31191 Cordovilla, Spain

**Keywords:** electrospinning, PVDF, corrosion, superhydrophobicity

## Abstract

Among the various polymeric options employed for the deposition of electrospun coatings, poly(vinylidene fluoride) (PVDF) has been widely investigated thanks to its excellent mechanical properties, high chemical resistance, and good thermal stability. In this work, the electrospinning technique is used for the fabrication of functional PVDF fibers in order to identify and evaluate the influence of the experimental conditions on the nanofiber properties in terms of optical transmittance, wettability, corrosion resistance, and surface morphology. Some of these properties can play a relevant role in the prevention of ice formation in aircrafts. According to this, a matrix of 4 × 4 samples of aluminum alloy AA 6061T6 was successfully coated by controlling two operational input parameters such as the resultant applied voltage (from 10 up to 17.5 KV) and the flow rate (from 800 up to 1400 µL/h) for a fixed polymeric precursor concentration (15 wt.%). The experimental results have shown a multilevel fiber-bead structure where the formation of a fiber mesh directly depends on the selected operational parameters. Several microscopy and surface analysis techniques such as confocal microscopy (CM), field emission scanning electron microscopy (FE-SEM), UV/vis spectroscopy, and water contact angle (WCA) were carried out in order to corroborate the morphology, transmittance, and hydrophobicity of the electrospun fiber composite. Finally, the corrosion behavior was also evaluated by electrochemical tests (Tafel curves measurement), showing that the presence of electrospun PVDF fibers produces a relevant improvement in the resultant corrosion resistance of the coated aluminum alloys.

## 1. Introduction

In nature, the surfaces of many plants, insects, and other animals can repel wetting due to water droplets [1]. Lotus leaf is the most famous example of natural hydrophobicity where its surface is highly non-wettable [2,3]. Other representative examples are the penguin Spheniscus humboldti, which live in the world’s coldest environments; they have a thick layer of feathers that prevent the penetration of cold sea water to the skin, and their corresponding feathers exhibit both excellent hydrophobicity and antiadhesion characteristics needed to repel water microdroplets [4]. When the values of water contact angle (WCA) are larger than 150°, the surfaces are classified as superhydrophobic [5,6,7]. In these superhydrophobic surfaces, the resultant deposited water droplets can remain in a nonwetting Cassie–Baxter state [8], resulting from low-surface energy compounds and air trapping inside the textured surface [9,10,11]. The trapped air minimizes the interaction between the water droplet and solid surfaces [12]. Thus, the energy barrier to the removal of water droplets from superhydrophobic surfaces is decreased. Consequently, a wide variety of industrial applications such as anti-icing [13], antibacterial [14], self-cleaning [15], or even corrosion resistance surfaces [16], among others, have been widely implemented. Specially, in the aeronautical applications, where atmospheric icing originating from supercooled droplets can cause serious problems to aircrafts like drift and drag, as well as catastrophic imbalances at high flying speed. Anti-icing surfaces play an important function to avoid undesired controllability consequences and corrosion side effects [17,18,19,20,21,22].

To date, different approaches have been reported to fabricate rough surfaces with variable microstructures, such as the chemical etching method [23], electrochemical deposition [24], sol–gel process [25], layer-by-layer assembly [26], and chemical vapor deposition [27], among others. However, a simple, cost-effective, highly versatile method and one-step fabrication process for the large-scale fabrication of superhydrophobic micro/nanofibers is electrospinning [28]. This technique can provide low surface energy as a function of the selected polymeric precursor, and even a highly rough structure caused by the hierarchical microstructures and nanostructures of micro/nanofibrous mat [29], which could show a similar effect to the penguins’ body feathers [4]. The morphology surface roughness and the structure of the electrospun mats can be affected by various parameters, such as the intrinsic polymeric precursor properties (concentration, molecular weight, viscosity, surface tension, and nature of solvent), the operational condition parameters (applied voltage, flow rate, and distance of tip to collector), and the environmental conditions (relative humidity and temperature) [30,31]. Because of their high surface and a specific fiber morphology that can be perfectly tuned using polymeric materials and the corresponding processing parameters, electrospun mats can present several advantages for different applications that involve contact with an aqueous fluid [32]. More specifically, adequate control of the previous parameters and the selection of a polymeric precursor with an intrinsic hydrophobic behavior by showing a low surface energy make an increase in the wettability properties possible. In this sense, an important advantage of electrospinning is the possibility of obtaining fibers with a high surface area to volume ratio, an aspect of great interest in industrial applications, which require superhydrophobicity. Furthermore, this technique also provides additional advantages such as easy-scalability and the possibility of obtaining multifunctional surfaces thanks to its great versatility [29].

Among the various electrospun polymer systems, poly(vinylidene fluoride) (PVDF) was selected in this work for the development of the electrospun fiber mats thanks to its excellent mechanical strength and thermal stability [33,34] combined with a very good corrosion resistance [35]. Another aspect to remark is that PVDF is an interesting candidate for the production of highly hydrophobic surfaces thanks to its favorable properties such as a low surface energy (25 dynes cm^−1^) and sound chemical inertness [36], which is associated with its chemical nature composed of alternating CH2 and CF2 functional groups, respectively [37]. In addition, PVDF can be also processed by the electrospinning technique, making possible a higher non-wettability property (superhydrophobic behavior), which is associated with the fabrication of rougher surfaces in comparison with a flat film deposition [38]. The novelty of this work is that an accurate identification of the electrospun samples in terms of surface morphology, wettability, corrosion resistance, and UV/vis optical transmittance was obtained as a result of applying different input parameters in the electrospinning process. In addition, the most representative factors, which directly affect the surface morphology (fiber diameter, fiber density, presence of beads, and surface roughness) were compared with the wettability, corrosion resistance, and optical transmittance to find the different relationships that define an optimal performance. Finally, the experimental results corroborate the important role of obtaining hierarchical fiber/beads composite and, in this way, the creation of micro-nano/multilevel structures for the optimization of superhydrophobic and anticorrosive surfaces.

## 2. Experimental Procedure

### 2.1. Reagents and Materials

Poly(vinylidene fluoride) (PVDF, (CH_2_CF_2_)*_n_*, Mw = 530,000 g/mol), dimethylformamide (DMF), and acetone were purchased from Sigma-Aldrich (Saint Luis, MO, USA). All reagents were used without any further purification. Firstly, the electrospun coatings onto standard glass slides (76 × 52 mm^2^, Sigma-Aldrich, Saint Louis, MO, USA) were analyzed to identify the characteristics of the morphology, wettability, and optical properties. Secondly, the corrosion properties were studied through electrospun fibers deposited onto aluminum alloy substrates, with a final dimension of 100 mm of length, 25 mm of width, and 2.5 mm of thickness, which were cut, polished with a roughness less than (S_a_ = 0.7248 µm; Ra = 0.6335 µm), and cleaned by means of acetone. Finally, this specific aluminum alloy (AA6061-T6) was selected as the reference substrate to test the corrosion resistance as it is one of the most important alloys used in aeronautical applications where a high mechanical strength and corrosion resistance are needed [39,40].

### 2.2. Electrospinning Procedure

In this work, PVDF was dissolved in a 3:2 solvents mixture of DMF and acetone (AC). Thus, a homogeneous PVDF solution of 15 wt % was obtained at room temperature and under stirring (200 rpm) for 12 h. Several works have analyzed the effect of different DMF/AC volume ratio and polymer concentration in the final solution viscosity [41,42,43,44].

The solution was loaded in to a 5 mL syringe located horizontally on a syringe pump; the distance between the capillary tip and the aluminum electrode (cathode) used as collector was 15 cm. The positive electrode (anode) for all the depositions was a 20-gauge needle with an inner diameter of 0.6 mm. The electrospun samples placed in the aluminum electrode were coated at room temperature (20 °C), at 40% humidity (RH), and for a deposition period of 20 min. Finally, the electrospinning experiments were conducted at various flow rates and applied voltages, which are shown in Table 1.

### 2.3. Characterization Techniques

The resultant surface morphologies of the electrospun PVDF coatings were analyzed using a confocal microscope (model S-mart, SENSOFAR METROLOGY, Barcelona, Spain). The confocal microscope applies a Corse Shift single algorithm with an objective of EPI 50X v35 for an area of 340.03 × 283.73 μm^2^. According to the standard ISO 25178, the surface roughness (S-L) measurements were obtained with three different filters: a low filter (F-operator-level), a high filter (S-filter, standard cut off λ_s_: 2.5 µm), and a Gaussian filter (L-filter, standard cut off λc: 0,08 mm, S_a_ < 0.02 µm). These topography measurements were used for estimating the average fiber diameter (D_f_) and surface roughness (S_a_) of the samples. Confocal microscopy was also employed for controlling the coating thickness by means step high measurements.

In addition, field emission scanning electron microscopy (FE-SEM, Hitachi S4800, Tokyo, Japan) was employed for a more exhaustive analysis of the topography measurements mentioned above.

Water contact angle (WCA) measurements were carry out with a CAM 100 contact angle goniometer (CAM 100, KSV Instruments, Burlington, VT, USA) using distilled water. The static water contact angle was measured three times and in six different places of the sample, which makes a total of 288 measurements. The results were calculated with the average and their average deviations of the measurements.

**Figure 1:**
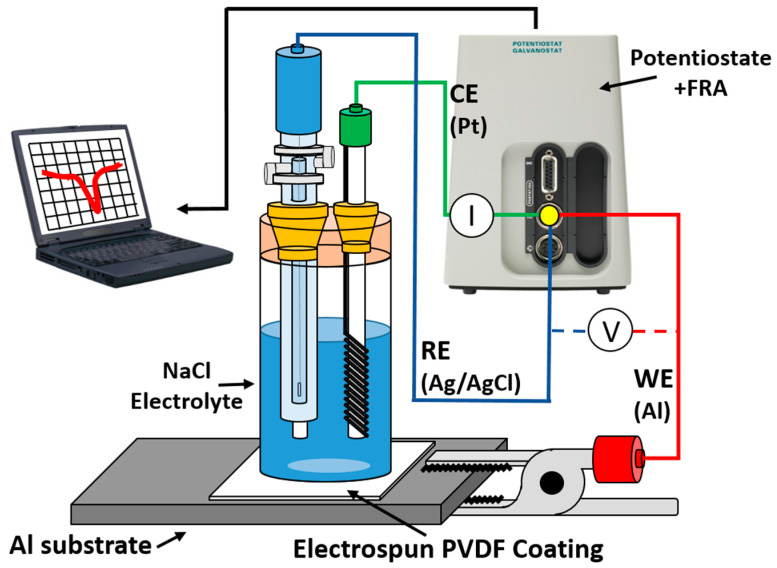
Tafel polarization test setup. PVDF, poly(vinylidene fluoride). Counter Electrode (CE), Reference Electrode (RE), Working Electrode (WE) and Frequency Response Analyzer (FRA).

Electrochemical measurements of Tafel polarization curves were carried out on an Autolab Potentiostat/Galvanostat PGSTAT302N (Metrohm, Herisau, Switzerland), according to the setup shown in Figure 1. All corrosion tests were performed at room temperature in 6 wt% NaCl aqueous solutions, using a conventional three-electrode cell consisting of a working electrode (bare or coated Al sample), a silver chloride Ag-AgCl reference electrode, and a platinum counter electrode. Before conducting all the experiments, the samples were immersed in the 6 wt% NaCl electrolyte for 30 min to make sure that the system is in steady state and with the open circuit potential (OCP) stabilized.

Tafel polarization measurements were obtained by scanning the electrode potential automatically from −200 mV to +200 mV with respect to the OCP voltage at a scan rate of 2 mV s^−1^. In addition to calculating the Tafel curves, it is very important to note the substrate properties (AA6061T6, density of 2.7 g/cm^3^, equivalent weight 26.98 g/mol, working electrode surface area of 2.01 cm^2^, and counter electrode surface area of 3.15 cm^2^).

The output from these experiments yielded a polarization curve of the current density versus the applied potential. The resulting corrosion current can be calculated using Tafel slope analysis, where it established a relationship between the current density and the electrode potential during the polarization. The corrosion data were obtained from Tafel polarization curves, where it was obtained by superimposing a straight line on the linear portions of the cathodic and anodic curves. Finally, other corrosion parameters, like equivalent weight of the metal, density, or exposed surface, are also required as input parameters. With this information, the software generates the complete set of corrosion parameters. Thus, the corrosion rate is calculated according to the following equation [45].

(1) $$\mathrm{Corrosion}\;\mathrm{Rate}=327\times \frac{i_{\mathrm{corr}}\;M}{V\cdot D\cdot \;A}\times 100\%$$

where 327 = 1 year (in seconds)/96,500, and 96,500 = 1 F in coulombs. I_corr_ is the corrosion current and is determined by an intersection of the linear portions of the anodic and cathodic sections of the Tafel curves, M is the atomic mass, V is the valence (number of electrons that are lost during the oxidation reaction), D is the density, and A is the exposed area of the sample.

The corrosion protection efficiency from Tafel polarization curves was calculated by the following formula [46,47]:

(2) $$\eta =\frac{i_{\mathrm{corr}}-i_{\mathrm{corr}}\left(C\right)}{i_{\mathrm{corr}}}\times 100\%$$

In this equation, i_corr_ and i_corr_(C) correspond to the corrosion current densities of bare aluminum and coated aluminum, respectively.

Finally, UV/vis spectroscopy was used to characterize the optical properties of the electrospun PVDF fibers. All optical transmittance spectra were carried out in the spectral range from 350 to 900 nm on the UV/vis spectrophotometer at room temperature. A Jasco V-360 spectrophotometer (Agilent, Santa Clara, CA, USA) was used to perform all the measurements.

## 3. Results and Discussion

### 3.1. Surface Morphology

**Figure 2:**
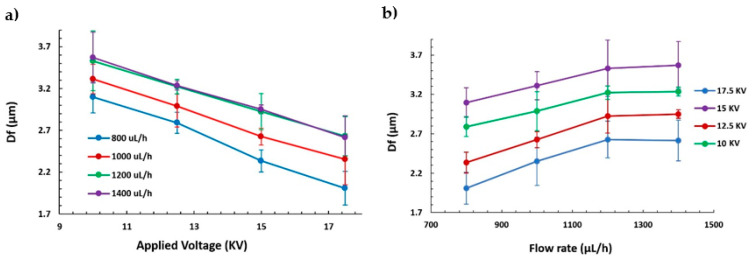
(**a**) Evolution of the fiber diameter (D_f_) as a function of the applied voltage (10, 12.5, 15, and 17.5 KV) at different flow rates (800, 1000, 1200, and 1400 µL/h). (**b**) Evolution of the fiber diameter (D_f_) as a function of flow rate (800, 1000, 1200, and 1400 µL/h) at several applied voltages (10, 12.5, 15, and 17.5 KV).

The final morphology of the electrospun samples was studied to understand the effect of varying these two specific parameters (applied voltage and flow rate). The samples were measured by confocal microscope and field emission scanning electron microscopy (FE-SEM) in several locations to obtain a representative number of measurements, in order to determine the average values and their average deviations, which are represented by the error bars. On one hand, one of the main characteristics of the morphology is the fiber diameter (see Figure 2), where 160 fibers were evaluated through the most representative 10 fibers per sample. Figure 2a clearly shows how the fiber diameter of the electrospun fibers is decreased when the applied voltage is increased for all the different fixed flow rates. Furthermore, in Figure 2b, the opposite effect can be appreciated, where an increase in the flow rate has produced an increase in the fiber diameter up to a maximum flow rate of 1200 µL/h, where the diameter is kept constant for a higher applied voltage.

**Figure 3:**
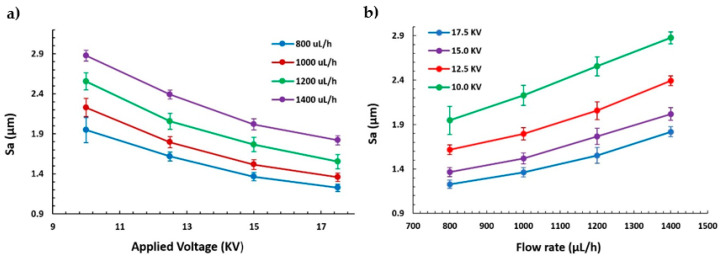
(**a**) Evolution of the average surface roughness (S_a_) as a function of the applied voltage (10, 12.5, 15, and 17.5 KV) at different flow rates (800, 1000, 1200, and 1400 µL/h). (**b**) Evolution of the average surface roughness (S_a_) as a function of flow rate (800, 1000, 1200, and 1400 µL/h) at several applied voltages (10, 12.5, 15, and 17.5 KV).

**Figure 4:**
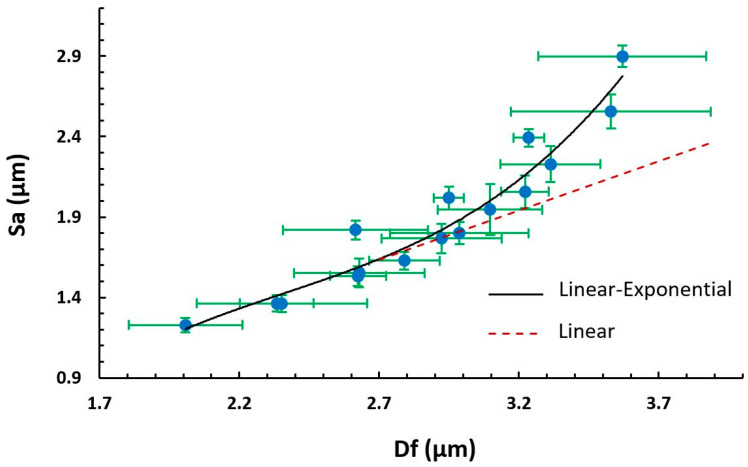
Evolution of the average surface roughness (S_a_) as a function of the fiber diameter (D_f_) from linear to exponential relationship owing to the influence of beads.

These results are in concordance with the literature, where previous works have demonstrated that a high voltage allows stretching forces capable of promoting the formation of distributed uniform fibers [37] and the effect of increasing the applied voltage produces thinner fibers owing to the production of a higher electrostatic repulsive force on the fluid jet [48,49]. Nevertheless, taking into account other works, an adequate selection of the voltage depends on the system used such as type of solvent and polymer concentration [50,51]. In addition, the effect of the flow rate is other parameter that can control the resultant fiber diameter and, for this reason, low feeding rates are desirable because the solvent has enough time for a complete evaporation before reaching the collector. In addition, high flow rates produce beaded fiber formation and an increase in the fiber diameter due to the unavailability of a proper drying time of the solvent when the collector is reached [52]. On the other hand, another important morphology characteristic is the average surface roughness (S_a_), as can be appreciated in Figure 3. Figure 3a,b show a similar behavior of S_a_ with respect to fiber diameter (D_f_), where an increase in the flow rate has produced a higher surface roughness. This phenomenon can be noticed in Figure 4, where there is a change in trend from linear to exponential relationship between the fiber diameter and the surface roughness. This change was generated as a result of an increment of the presence of beads in the protrusion of the fiber surface, as can be further observed in the confocal and scanning electron microscopy (SEM) images.

**Figure 5:**
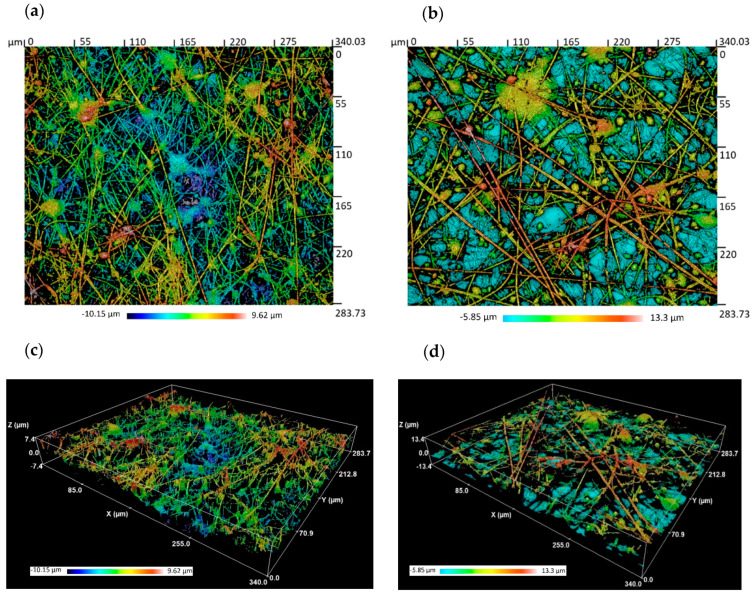
Confocal microscope 2D/3D images representing the surface morphology of the electrospun mats for sample S1 (**a**,**c**) (800 µL/h and 17.5 KV) and sample S16 (**b**,**d**) (1400 µL/h and 10 KV).

**Figure 6:**
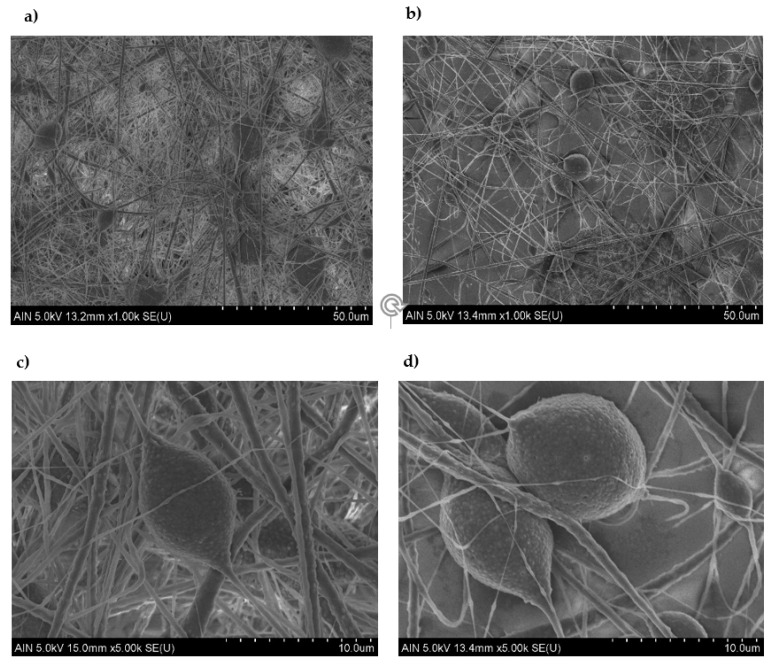
Scanning electron microscopy (SEM) images representing the surface morphology of the electrospun mats for sample S1 (**a**,**c**) (800 µL/h and 17.5 KV) and sample S16 (**b**,**d**) (1400 µL/h and 10 KV) in the scale of 50 µm (**a,b**) and 10 µm (**c,d**) respectively.

In order to understand how the influence of beads takes place, some of the samples with lower and higher surface roughness were analyzed. The confocal and SEM images presented in Figure 5 and Figure 6 show a dense structure of entangled threads, beads, and droplets. However, clear differences were observed. On one side, the lower surface roughness images represented in Figure 5a and Figure 6a,c, which correspond to the higher applied voltage and the lower flow rate, exhibit a low number of beads entangled in a high density mesh of thin fibers. On the other side, in Figure 5b and Figure 6b,d, where the surface roughness is increased (low applied voltage and an increment of the flow rate), the influence of beads gains more relevance by the loss of the fiber density in combination with the presence of a higher number of beads and a larger fiber diameter.

On the other hand, concerning the thickness control, it is important to note that we are dealing with a particular kind of “fluffy” coating instead a compact layer, which means that, on one hand, the thickness is not so well defined as in a conventional coating and, on the other hand, the role of the layer as a barrier, for chemical and optical purposes, is affected not only by the thickness, but also by other parameters such as compactness, number, size, and distribution of pores, size of fibers, and so on. The step high measurements show great differences, from 2 to 55 microns, as can be seen in Table 2. If we were dealing with compact coatings, we could expect smaller differences, proportional to the flow rates (1400 to 800), but the reality is that the final thickness is much more influenced by the combination of operational parameters (flow and voltage), with the voltage being the most relevant.

### 3.2. Wettability Properties

The study of the wettability properties in combination with the characterized surface morphology implies an interesting research point.

**Figure 7:**
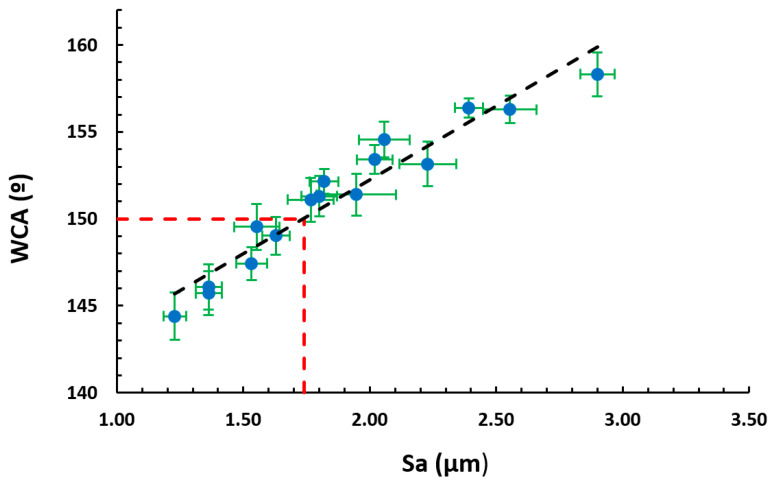
Linear relationship of the static water contact angles (WCAs) as a function of the average surface roughness (S_a_). The samples with a surface roughness (S_a_) higher than 1.74 µm present a superhydrophobic behavior (WCA > 150°).

The experimental results presented in Figure 7 show a liner relationship between the WCA and the average roughness (S_a_). According to the diagram, a higher roughness resulted in higher contact angles and a decrease in the wettability, which is desired.

When a water droplet rests on a solid surface, a physicochemical balance is generated. This balance state is driven by the chemistry and the geometrical structure of the surface. On one side, the surface chemistry interacts through three interface free energies (liquid–gas, solid–gas, and solid–liquid), which are in a thermodynamics balance. These balanced free energies, which are explained by the Young’s equation, determine the shape of the droplets though the equilibrium water contact angle (EWCA) in an ideal smooth surface; see Equation (3).

(3) $$\cos\theta =\frac{\gamma_{SG}-\gamma_{SL}}{\gamma_{LG}}$$

where $\gamma_{LG},\gamma_{SG},\gamma_{SL}$ represent the surfaces interfaces free energies (N/m) from liquid–gas, solid–vapor, and solid–liquid respectively. In addition, *θ* is the EWCA for a smooth surface [53].

Therefore, to obtain a superhydrophobic surface (*θ* > 150°), it is important to reduce the surface free energies by the functionalization of low surface energy materials. In this case, the PVDF coating has low values of interfacial free energies due to the presence of fluoride functional groups [54]. Thus, smooth PVDF coatings have a hydrophobic behavior (*θ* > 90°).

On the other side, the geometrical structure of the surface plays an important role. This is the reason the Young model, which is applied for smooth surfaces, is a poor model to determine the behavior with a certain surface roughness. Thus, in order to understand the influence of rough surfaces, some different models have been reported. The most cited and highly criticized are the Wenzel and the Cassie–Baxter models [55,56], although transitions between both models are also reported [57]. In this work, the Cassie–Baxter model was selected to explain the wetting surface state due to the presence of air entrapped inside the gaps of the fibers net, which makes the penetration of the liquid into the roughness or texture of the surface difficult because of capillary forces. In this way, the Wenzel assumption can be breached [8]. As a result, the water droplets sit upon solid and air gaps, and do not follow the surface contours. The Cassie–Baxter equation for the solid–liquid–air composite interface (porous) considers the effect of air gaps under the droplets, as can be observed in Equation (4).

(4) $$\cos\theta_{obs}=f_{s}\left(\cos\theta +1\right)-1$$

where $f_{s}$ is the solid surface fraction (%); *θ* is the EWCA for a smooth surface, which is described in Equation (3); and *θ_obs_* is the observed static water contact angle (WCA). Thus, the WCAs of several samples were measured.

With the purpose of proving the experimental results presented in Figure 7, Equation (4) was analyzed. Considering that the term cos *θ* + 1 is a constant value, if the solid surface fraction decrease to zero, the WCA increases to 180°. This means that the increase in the average roughness reduces the area of contact between the liquid and solid and results in a reduction in the adhesion of a droplet to the solid surface.

**Figure 8:**
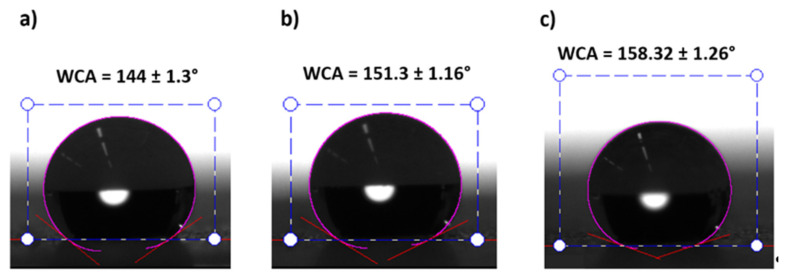
Static water contact angles (WCAs) of samples S1 (**a**), S7 (**b**), and S16 (**c**). Sample S1 (**a**) exhibits a hydrophobic behavior (90° < WCA < 150°) and samples S7 (**b**) and S16 (**c**) exhibit a superhydrophobic behavior (WCA > 150°).

According to this, the improvement in the wettability properties of the electrospun fibers mats is mainly caused by the multilevel roughness of the surface, which is induced by micro/nano-sized fibers combined with the presence of beads, due to the loss of the fiber density [58]. This hierarchical structure with micro and nano roughness generates a substantial increase in air gaps, which leads to superhydrophobic surfaces (*θ_obs_* > 150°) [59,60]. As can be seen in Figure 7, all the samples with a surface roughness (S_a_) higher than 1.74 µm present a superhydrophobic behavior and the rest are hydrophobic (90° < *θ_obs_* < 150°). The most representative samples are presented in Figure 8.

### 3.3. Optical Properties

Optical properties such as transmittance are interesting data for several reasons. For instance, the presence of water droplets on the coated surface can be detected owing to a variation in the refractive index [61], which can be measured via optical fiber sensor devices, provided we can have control of the absorption of light due to the coating. In addition, a measure of the transmittance allows us an easy-to-implement way of controlling the coating thickness, which is highly dependent on the process parameters.

**Figure 9:**
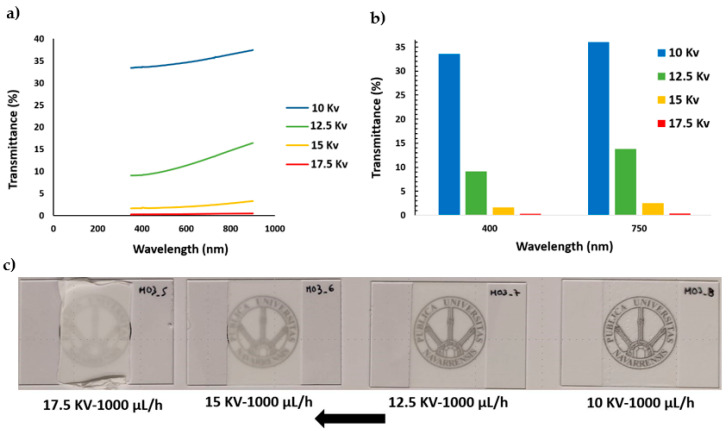
(**a**) UV/vis spectra of the resultant PVDF fibers for a fixed flow rate (1000 µL/h) and different applied voltage (10, 12.5, 15, 17.5 KV); (**b**) transmittance change at a specific wavelength of the visible region at 400 nm and 750 nm; (**c**) electrospun samples S5, S6, S7, and S8 appearance onto standard glass slides.

**Figure 10:**
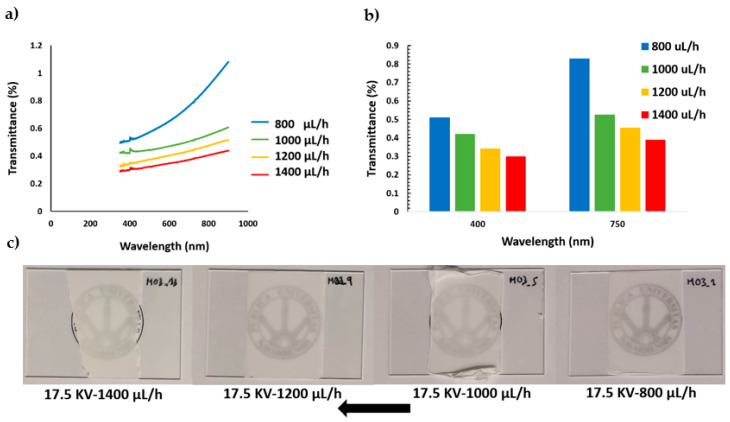
(**a**) UV/vis spectra of the resultant PVDF fibers for a fixed voltage (17.5 KV) and different flow rates (800, 1000, 1200, 1400 µL/h); (**b**) transmittance change at a specific wavelength of the visible region at 400 nm and 750 nm; (**c**) electrospun samples S1, S5, S9, and S13 appearance onto standard glass slides.

UV/vis spectra of all PVDF samples were analyzed in the wavelength range of 350–800 nm. In addition, with the aim of highlighting the process parameter dependence, two aspects were considered in the transmittance representations, as can be appreciated in Figure 9 and Figure 10, respectively. On one side, Figure 9a presents the influence of keeping a fixed flow rate for different applied voltages and, on the other side, Figure 10a shows the effect of applying several flow rates for a fixed applied voltage. The experimental results indicate that an increase in the applied voltage from 10 KV to 17.5 KV at a fixed flow rate (1000 µL/h) produces a decrease in the light transmittance property. This effect can be clearly appreciated in Figure 9b, where the light transmittance of the PVDF samples decreased from 33.57% to 0.30% and 36% to 0.41% for 400 and 750 nm, respectively.

On the other hand, the experimental results also show a similar tendency when the flow rate is changed from 800 µL/h to 1400 µL/h at a fixed applied voltage (17.5 KV), although this variation is not too prominent in comparison with the change in the applied voltage. This effect can be appreciated in Figure 10b, where the transmittance only decreases from 0.51% to 0.30% and 0.83% to 0.38% for 400 and 750 nm, respectively. Therefore, the reduction in the optical transmittance due to an increment of the applied voltage (Figure 9a) is significantly higher than the effect of increasing the flow rate (Figure 10a). This is the reason the transparency change of the samples can be seen clearly in Figure 9c and hardly in Figure 10c.

As could be expected, samples with the same thickness have comparable values of transmittance. These relatively slight differences in transmittance must be related to the diffusion of light phenomena, which can be directly related to the coating thickness and density of the fiber mesh, which becomes more opaque for higher voltages and flows that correspond to thicker coatings [62].

This fact might seem not too relevant, but we would like to insist that this investigation is carried out in the frame of an applied research project that aims to develop industrial coatings. In this sense, we have chosen flow and voltage as the main parameters, comparing the results obtained in the same deposition time. Light transmittance is, in this scheme, the basis of an easy to implement system of process control.

### 3.4. Anticorrosion Performance

**Figure 11:**
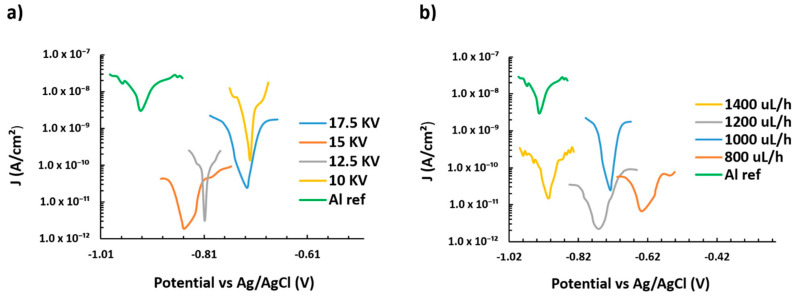
Tafel plots corresponding to the aluminum bare substrate and the different aluminum samples coated by PVDF electrospun fibers mats after being tested in 6 wt % NaCl aqueous solution; (**a**) electrospun samples S1, S5, S9, and S13 for a fixed flow rate (1000 µL/h) and different applied voltages (10, 12.5, 15, 17.5 KV), respectively; (**b**) electrospun samples S5, S6, S7, and S8 for a fixed voltage (17.5 KV) and different flow rates (800, 1000, 1200, 1400 µL/h), respectively.

Before starting the corrosion tests of the coated samples, the bare aluminum substrate (AA6061T6) corrosion behavior was measured as a reference to be later compared to the results from the different samples. In addition, with the aim of simplifying the obtained results as in the optical study, two aspects were considered for the Tafel polarization curves. In Figure 11, the influence of keeping a fixed flow rate is presented for different applied voltages (Figure 11a) as well as the effect of applying several flow rates for a fixed applied voltage (Figure 11b). All these results are summarized in Table 3.

In typical Tafel polarization curves, the excellent corrosion resistance always possesses a lower corrosion rate (CR), which corresponds to a higher corrosion potential (E_corr_) or a lower corrosion current density (J_corr_) [62,63,64,65].

The corrosion results show that all the electrospun samples minimize the corrosion current density and the corrosion rate of the aluminum substrate in three orders of magnitude, leading to a considerable improvement in the resultant protection efficiency. Furthermore, Table 3 indicates that an increase in the applied voltage from 10 KV to 17.5 KV at a fixed flow rate (1000 µL/h) involves a decrease of the corrosion current density (J_corr_), a lower corrosion rate, and an enhancement of the protection efficiency (η). However, when the flow rate is changed from 800 µL/h to 1400 µL/h at a fixed applied voltage (17.5 KV), this results in an increase of the corrosion current density (J_corr_), a reduction of the corrosion potential (E_corr_), a higher corrosion rate, and a loss of the protection efficiency (η). The best results were found to sample S1, which shows the lowest corrosion current density and corrosion rate in comparison with the other samples.

**Figure 12:**
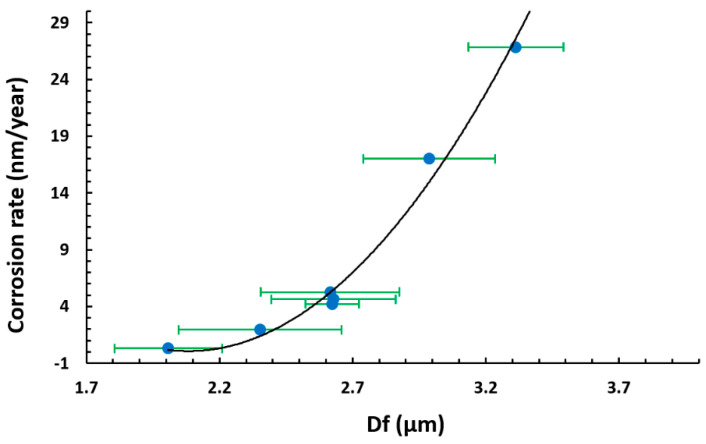
Exponential behavior of the corrosion rate as a function of the fiber diameter (D_f_).

Whereas the coating thickness seems not to play a relevant role in this behavior, the relationship between the fiber diameter and the corrosion rate shows a clear correlation, as shown in Figure 12. The samples with a lower value of fiber diameter, which is related to a greater polymeric active surface due to higher density mesh of thin fibers and the presence of lesser number of bead, exhibit a higher reduction in the corrosion rate.

## 4. Conclusions

The study of PVDF electrospun coatings deposited at different voltages and flow rates has shown how the combination of these parameters plays an important role in determining the characteristic of the surface morphology and performance. The effect of increasing the applied voltage produces thinner fibers, whereas higher flow rates produce beaded fiber formation and an increase in the fiber diameter. In addition, there is a change in trend from a linear to exponential relationship between the fiber diameter and the surface roughness due to an increment of the presence of beads in the protrusion of the fiber surface.

In this way, hierarchical fiber/beads’ surfaces (corroborated by confocal microscope and SEM) were obtained, showing a superhydrophobic behavior (WCA higher than 150°), mainly caused by the multilevel roughness of the surface, which is induced by micro/nano-sized fibers combined with the presence of beads, due to the loss of the fiber density.

In addition, the corrosion protection of the substrate changes as a function of the morphological features. The samples with a smaller fiber diameter, related to a higher density mesh of thin fibers and a greater active surface, exhibit a higher reduction of the corrosion rate.

On the other side, an increase in the applied voltage produces a decrease in the light transmittance property in a significant way, in contrast with an increase of the flow rate, where there are insignificant changes. This allows to monitor the changes in coating thickness, which are mainly related to the employed voltage.

Finally, the results obtained by this study can suggest a starting point to identify and optimize the operational parameters in specific fixed conditions for high-performance wettability and/or corrosion resistance specially in aeronautical applications, among others.

## Figures and Tables

**Table 1:** Summary of the operational inputs parameters for the fabrication of the electrospun coatings as a function of the flow rate and applied voltage, respectively.

Sample	Flow Rate (µL/h)	Voltage (KV)
S1	800	17.5
S2	800	15
S3	800	12.5
S4	800	10
S5	1000	17.5
S6	1000	15
S7	1000	12.5
S8	1000	10
S9	1200	17.5
S10	1200	15
S11	1200	12.5
S12	1200	10
S13	1400	17.5
S14	1400	15
S15	1400	12.5
S16	1400	10

**Table 2:** Summary of coating thickness measured by confocal microscopy using the step high technique.

Sample	Voltage (KV)	Flow Rate (µL/h)	Thickness (µm)
S8	10	1000	2.3 ± 0.2
S12	10	1200	5.2 ± 0.2
S3	12.5	800	5.5 ± 0.5
S7	12.5	1000	12.8 ± 1.5
S11	12.5	1200	26.3 ± 1.1
S6	15	1000	26.7 ± 0.6
S1	17.5	800	27.5 ± 0.6
S5	17.5	1000	36.3 ± 0.6
S14	15	1400	41.1 ± 1.0
S9	17.5	1200	42.2 ± 1.3
S13	17.5	1400	55.5 ± 2.6

**Table 3:** Summary table of the Tafel analysis for uncoated aluminum substrate (6061T6) and poly(vinylidene fluoride) (PVDF) electrospun coatings of all the samples of this study after being tested in 6 wt % NaCl aqueous solution. β is the reaction’s Tafel constant (constant for a given reaction, with units of volts/decade. βa is the anodic β Tafel constant in volts/decade and βc is the cathodic β Tafel constant in volts/decade.

Sample	Voltage (KV)	Flow Rate (µL/h)	J_corr_ (nA/cm²)	E_corr_ (V)	Corrosion rate (nm/year)	βa (V/dec)	βc (V/dec)	η (%)
Al	0	0	620.35	−0.925	6312.3	3.6516	1.8372	0
S1	17.5	800	0.029	−0.635	0.298	0.236	0.144	99.995
S5	17.5	1000	0.191	−0.744	1.942	0.088	0.029	99.969
S9	17.5	1200	0.455	−0.787	4.626	0.199	0.188	99.927
S13	17.5	1400	0.517	−0.917	5.264	0.475	0.714	99.917
S5	17.5	1000	0.191	−0.744	1.942	0.088	0.029	99.969
S6	15	1000	0.416	−0.821	4.228	1.250	4.289	99.933
S7	12.5	1000	1.673	−0.810	17.018	0.661	2.180	99.730
S8	10	1000	2.638	−0.725	26.839	0.035	0.066	99.575

## Data Availability

Not applicable.
